# Spindle assembly checkpoint: the third decade

**DOI:** 10.1098/rstb.2011.0072

**Published:** 2011-12-27

**Authors:** Andrea Musacchio

**Affiliations:** 1 Department of Experimental Oncology, European Institute of Oncology, Via Adamello 16, 20139 Milan, Italy; 2 Max Planck Institute of Molecular Physiology, Otto-Hahn-Strasse 11, 44227 Dortmund, Germany

**Keywords:** spindle assembly checkpoint, kinetochore, cell cycle, Aurora B, KMN network

## Abstract

The spindle assembly checkpoint controls cell cycle progression during mitosis, synchronizing it with the attachment of chromosomes to spindle microtubules. After the discovery of the *mitotic arrest deficient* (*MAD*) and *budding uninhibited by benzymidazole* (*BUB*) genes as crucial checkpoint components in 1991, the second decade of checkpoint studies (2001–2010) witnessed crucial advances in the elucidation of the mechanism through which the checkpoint effector, the mitotic checkpoint complex, targets the anaphase-promoting complex (APC/C) to prevent progression into anaphase. Concomitantly, the discovery that the Ndc80 complex and other components of the microtubule-binding interface of kinetochores are essential for the checkpoint response finally asserted that kinetochores are crucial for the checkpoint response. Nevertheless, the relationship between kinetochores and checkpoint control remains poorly understood. Crucial advances in this area in the third decade of checkpoint studies (2011–2020) are likely to be brought about by the characterization of the mechanism of kinetochore recruitment, activation and inactivation of checkpoint proteins, which remains elusive for the majority of checkpoint components. Here, we take a molecular view on the main challenges hampering this task.

## 1. Preamble

This essay aims to discuss a handful of crucial practical and conceptual problems facing future studies in the area of spindle assembly checkpoint (SAC) signalling. Admittedly, the content is speculative and reflects the author's own biases in the interpretation of a rather large body of solid facts and an equally large collection of contradictory observations. While trying not to ignore known facts and observations, the review avoids lengthy and detailed descriptions of checkpoint mechanisms, unless strictly necessary. Such mechanisms have been discussed in several recent reviews [1–5].

## 2. The spindle checkpoint in a nutshell

The SAC is a feedback control mechanism whose activity ensures accurate chromosome segregation during mitosis. As with many other functions in the cell, the practical implementation of the checkpoint is entrusted to a set of dedicated ‘checkpoint proteins’. Well-characterized checkpoint components include the kinases Bub1, BubR1 and Mps1 [1]. Three additional kinases, Prp4, Chk1 and Tao1 have also been proposed to take part in the checkpoint [6–8]. At least in the case of Tao1, however, initial claims of a role in the checkpoint were recently put into question [9,10]. Other checkpoint components include Mad1, Mad2 and the three subunits of the Rod, Zwilch and ZW10 (RZZ) complex. These proteins are (at least in a broad sense) devoid of catalytic activity and are implicated in protein–protein interactions [1]. Together, the checkpoint proteins contribute to the formation of a checkpoint effector complex, named the mitotic checkpoint complex (MCC) [11–13]. The MCC protects Cyclin B and Securin from ubiquitination, preventing their destruction by the proteasome, and thus maintaining the mitotic state.

The Cdk1 : Cyclin B complex is the ‘engine’ of cell cycle progression [14]. It generates the thrust that sets into motion the ‘wheels’ associated with mitosis, including nuclear envelope breakdown, assembly of the mitotic spindle, chromosome condensation, and so on [15]. The SAC supports retrograde feedback signalling to the cell cycle engine from one of the spinning wheels, the process of attachment of chromosome to the spindle [16]. In essence, the SAC prevents the degradation of Cyclin B and Securin until completion of the process of attachment of chromosomes to spindle microtubules (metaphase) [17,18]. The checkpoint's services render metaphase an obligatory intermediate of mitosis. This is because Cdk1 : Cyclin B activity continues to be delivered in each and every cell containing a functional checkpoint precisely for the amount of time required for achieving metaphase. In the absence of checkpoint control, human cells leave mitosis prematurely with unattached or improperly attached chromosomes, usually encountering a disastrous fate [19]. Under certain circumstances, the resulting imbalances in chromosome composition (aneuploidy and polyploidy) might also result in cellular transformation, in particular if occurring concomitantly with loss of function of genomic gatekeepers [20].

The kinetochore is the main site of attachment of microtubules to mitotic chromosomes. The kinetochore's ‘hardware’ is made of a core of approximately 30 evolutionarily conserved scaffold proteins that create a solid link between chromatin and the mitotic spindle [21–24]. The ‘software’ is provided by a number of regulatory proteins that become recruited to kinetochores and are regulated there to deliver their activity as appropriate [2,22,25,26]. Among these are the components of the SAC, all of which are recruited to kinetochores during mitosis [1].

There are several accounts of kinetochore-independent contributions to checkpoint signalling of the checkpoint components [11,13,27–32]. Nevertheless, it is now established beyond reasonable doubt that kinetochore integrity is essential for checkpoint function: ablation of crucial components of the kinetochore hardware impairs the recruitment of the checkpoint components and results in a checkpoint defect (as discussed more thoroughly below) [19,33]. Indeed, kinetochores might behave as catalysts in the production of the checkpoint effector, the MCC [11–13]. Kinetochores might be accelerating specific rate-limiting steps in this process [5,34–36].

## 3. The sensory apparatus of the spindle assembly checkpoint

Understanding how kinetochores bind microtubules and regulate checkpoint status accordingly is the major challenge for mitotic checkpoint studies in the next decade. A crucial question regards what is being sensed by the checkpoint and how. Much of the discussion on the sensory apparatus of the SAC in the last decade has taken place within the rather fuzzy boundaries of the tension versus attachment dichotomy [37–42]. Recent faint glows indicate that the field is becoming ripe for a modern synthesis bringing about the substitution of these rather slippery concepts with more precise molecular descriptions and models. What are the crucial challenges confronting the community?

Pioneering experiments by Nicklas and colleagues in the late 1960s demonstrated that tension stabilizes kinetochore–microtubule attachment, and that an error-correction mechanism discriminates between correct and incorrect attachments, selectively destabilizing the latter [43]. The experiments hinted at the existence of a tension sensor, but of course did not clarify its location or molecular nature. Indeed, over 20 years later, these questions remained the object of interesting speculation [44]. In the mid-1990s, it became clear that kinetochores and centromeres might be crucially involved in the detection of tension [45–49]. This initiated a search for the molecular components of the tension-sensitive pathway. Experiments in the early 2000s in *Saccharomyces cerevisiae* brought to light a role of the essential centromere and kinetochore serine/threonine kinase Ipl1/Aurora B in the correction of improper kinetochore–microtubule attachments [50–52]. When the activity of this kinase is inhibited, improper and hyperstable kinetochore–microtubule attachments accumulate in high numbers, possibly suggesting that the process of error correction is inhibited.

With the discovery of the checkpoint genes [53,54], an essential intellectual challenge was readily recognized. Was there a relationship between the checkpoint and the error-correction mechanism exposed by Nicklas and colleagues, and if so, which one [44]? Ten years later, the observation that Aurora B is primarily required to sustain the SAC under conditions believed to reduce tension at the kinetochore microtubule interface, but not upon microtubule disassembly (i.e. not under conditions of lack of microtubule attachment) [42,55–61] led to the formulation of a popular model to describe the possible connection between error correction and the checkpoint [42,58]. In this model, lack of tension activates error correction, which in turn generates unattached kinetochores (as end-products of the correction activity) that stimulate the SAC response. The tension versus attachment dichotomy embeds the idea that the checkpoint is exclusively interested in lack of attachment, and becomes satisfied by any type of microtubule attachment, even of the type that cannot generate tension (for recent discussions see [37,38–42]). It is only because of the Aurora B-dependent activity of the error-correction mechanism, and the subsequent creation of unattached kinetochores, that the checkpoint becomes re-activated at a kinetochore after microtubule binding. The model predicts that if error correction is inhibited, e.g. through the inhibition of Aurora B, the checkpoint becomes automatically satisfied.

The model discussed in the previous paragraph assumes that the SAC and error-correction machineries are distinct, i.e. that they have a distinct molecular composition. Indeed, checkpoint proteins like Mad1 and Mad2 do not influence the state of kinetochore–microtubule alignment. With one notable recently reported exception [62], there is no evidence that these proteins participate in error correction or more generally in kinetochore–microtubule attachment [63–65]. For most other checkpoint proteins, on the other hand, a role in kinetochore–microtubule attachment is well established. In *S. cerevisiae*, most checkpoint components are not essential for viability [53,54,66], presumably because their services are not as important for faithful chromosome segregation as are those of *bona fide* error-correction components, such as Ipl1, which is encoded by an essential gene. However, loss of Bub1 and Bub3 results in highly increased rates of chromosome segregation errors [63–65]. In mammalian cells, *bona fide* SAC components such as Mps1, Bub1 and BubR1 appear to be involved in chromosome congression and error correction [65,67–69] in a way that is hardly distinguishable from that of Aurora B, at least on the basis of the assays currently available to monitor error correction.

These observations raise questions regarding the actual role of Aurora B in the checkpoint. If bona fide SAC kinases are also involved in error correction, cannot Aurora B be implicated in the SAC? Is the role of Aurora B in the SAC merely indirect, or rather does Aurora B participate directly in it? Support for a direct role of Aurora B in checkpoint signalling from unattached kinetochores has been gathered in several systems, including budding and fission yeast, frogs and humans [36,70–76]. In one recent study, Aurora B activity was shown to be essential for checkpoint signalling under conditions of complete or at least near-complete microtubule depolymerization, i.e. under conditions in which the checkpoint cannot be satisfied by residual microtubules [74]. In another study, activity of Aurora B was shown to sustain the checkpoint-dependent metaphase arrest observed upon constitutive targeting of the Mad1 : Mad2 complex to kinetochores [36]. In the latter case, the Aurora B-dependent metaphase arrest was shown to be compatible with the presence of a full complement of kinetochore microtubules, i.e. it occurred in the absence of unattached kinetochores or of clearly recognizable products of error correction [36].

The significance of these important findings is that activity of Aurora B might be directly implicated in checkpoint signalling, independently from its established function in error correction. Furthermore, evidence that Aurora B controls kinetochore recruitment of most checkpoint components (in the absence of microtubules, i.e. under conditions of lack of attachment), including Mps1 [30,55,69,77,78], and that it is important for their phosphorylation [74] suggests that it might act near (or at) the apex of the checkpoint signalling pathway. Furthermore, inhibition of Aurora B does not result in overt kinetochore assembly defects [74,79]. This suggests that factors such as the disappearance of crucial phospho-epitopes or the impairment of specific conformational changes, rather than gross changes in kinetochore composition, might be responsible for impaired kinetochore recruitment of the checkpoint proteins when Aurora B is inhibited.

Tracking the precise role of Aurora B and other mitotic kinases is an important prelude to addressing the fundamental question of what type of offenses are sensed by the checkpoint and error-correction pathways and how and where they are sensed. If tension- and attachment-dependent signalling were distinct, one would have to postulate the existence of two distinct sensory apparatuses. On the other hand, there might be a single sensory apparatus, concomitantly eliciting error correction and spindle checkpoint signalling [44]. Occam's razor, admittedly a rather blunt tool, makes us inclined towards the latter hypothesis. From a biochemical perspective, this hypothesis is far simpler because it predicts that the same sensor concomitantly elicits the activation of chromosome alignment/error correction and spindle checkpoint signalling pathways that share—before diverging—several components, most notably the Mps1 kinase and probably also the Bub1 and BubR1 kinases. If the sensors were distinct, and yet the pathways they activate shared at least a subset of their components, one would have to imagine circumstances in which the pathways would have to operate independently. Biochemically, this would imply directing the activity of the shared components (e.g. Mps1 kinase activity) towards different substrates depending on which pathway is active. This appears unlikely, given that both error correction and spindle checkpoint signalling occur at kinetochores and that this is where the checkpoint and error-correction components localize.

If there is a single sensory apparatus for error correction and SAC signalling, then previous results indicating that Aurora B is dispensable for the checkpoint response to unattached kinetochores might be the consequence of incomplete loss of function. Future studies will have to address this possibility. Indeed, there is evidence that a very low level of Aurora B activity is sufficient to maintain the checkpoint in the presence of unattached kinetochores. For instance, checkpoint inactivation at high doses of microtubule-depolymerizing agents is achieved only with relatively high doses of the Aurora B inhibitors hesperadin or ZM447439 [74]. However, the deleterious effects of Aurora B inhibition on checkpoint status are strongly synergic with concomitant inhibition of Bub1 or Mps1 [74,80].

## 4. The kinetochore

The arguments built in the previous section lead to the conclusion that it is essential to understand error correction and spindle checkpoint signalling in the context of the complex structural organization of the kinetochore. A complete account of kinetochore organization is beyond the scope of this review and several reviews on this topic have been made available recently [21,22]. Suffice to say that kinetochores are thought to consist of an inner plate hosting an interface between the 15-subunit constitutive centromere-associated network (CCAN) and specialized centromeric chromatin; and an outer plate containing the 10-subunit KMN network (from the initials of its Knl1, Mis12 and Ndc80 subcomplexes) implicated in microtubule binding. The CCAN and KMN networks are tightly connected. An interaction between CENP-C (CCAN) and the Mis12 complex (KMN network) provides an important point of contact [81–83]. The existence of additional points of contact, including one between CENP-T (CCAN) and the Ndc80 subcomplex (KMN network) [83,84], is supported by extensive analyses of localization dependencies of kinetochore proteins and from initial experiments of biochemical reconstitution.

Biochemical and structural studies, including super-resolution investigations of kinetochore organization by fluorescence microscopy, have finally resulted in rather precise maps of the relative position of many structural kinetochore proteins and of several checkpoint components [85–88]. For instance, these studies clarified that the highly elongated 4-subunit Ndc80 complex, conserved in all eukaryotes, orients its approximately 60 nm long axis at a relatively small angle with the inter-kinetochore axis [85–87]. The kinetochore-binding end of the Ndc80 complex was predicted to be positioned near the Mis12 and Knl1 complexes [87,88], and this prediction was fully confirmed in experiments of biochemical reconstitution [89].

In prophase *Drosophila melanogaster* S2 cells, the C-terminal region of the Ndc80 subunit is located approximately 65 nm outward (i.e. towards the microtubule) relative to CENP-A, the histone H3 variant that marks centromeres from yeast to humans [40]. Remarkably, it was shown that this distance increases to approximately 100 nm when kinetochores experience microtubule-dependent tension at metaphase [40]. This phenomenon is now known as intra-kinetochore stretch or tension [40,90]. A major task for the future is to understand how intra-kinetochore stretch is generated when microtubules bind the kinetochore. At one extreme, intra-kinetochore stretching might be the result of a discreet conformational change in the kinetochore caused by microtubule binding [22,91]. While this is possible, it seems unlikely when considering the great structural complexity of kinetochores. Each microtubule-binding site contains six to eight copies of the approximately 30 structural kinetochore components [85]. Furthermore, in most species, kinetochores are designed to bind multiple microtubules. Plausibly, each microtubule-binding site becomes engaged at a different time. Thus, it may be more sensible to interpret intra-kinetochore stretching as a progressive, continuous distortion of the kinetochore when microtubules are added. In agreement with this idea, intermediate levels of stretching are observed when microtubule end dynamics are inhibited through addition of taxol [40].

Importantly, intra-kinetochore stretch was shown to correlate with the status of checkpoint activation, with high stretch being correlated with checkpoint satisfaction. This idea of checkpoint control is alternative to a previous idea that tension might be monitored at the centromere, i.e. between sister kinetochores (inter-kinetochore tension) [46]. A significant theoretical limitation of the idea that the checkpoint sensor monitors tension between sister kinetochores is that it is not applicable to meiosis I, when the sisters co-orient and the homologues pair through the chiasmata. Conversely, the intra-kinetochore tension idea is also applicable to checkpoint control in meiosis I.

## 5. Mechanism of checkpoint signalling

As a summary so far, kinetochores can be thought of as nano-sensors capable of sensing relatively small structural changes in their organization. The scale of these conformational changes is in range with the dimensions of several kinetochore components. As already indicated above, the long axis of the Ndc80 complex is approximately 60 nm, while the Mis12 complex has a long axis of approximately 23 nm [89,92,93]. The length scale of these molecules is comparable with the approximately 35 nm increase in the distance between CENP-A and the C-terminal region of Ndc80 when kinetochores become attached.

How are these relatively small internal structural changes in kinetochores translated into robust checkpoint activity states? If Aurora B is indeed the primary checkpoint sensor, how does it sense conformational change within kinetochores? Several crucial recent observations started casting new light onto the mechanism through which Aurora B controls phosphorylation of its substrates. Aurora B is part of a larger complex, known as chromosome passenger complex (CPC), which contains Survivin, Borealin and inner centromere protein (INCENP) as additional subunits [94]. The CPC interacts with specialized centromeric chromatin that displays phosphorylated Thr3 on histone H3, a mark created by the kinase Haspin, as well as additional molecular beacons, such as phosphorylated histone H2A [95–99]. When placed at different depths within the kinetochore, a fluorescence resonance energy transfer sensor designed to be sensitive to Aurora B activity becomes differentially phosphorylated depending on the state of attachment [100]. When positioned near the kinetochore–centromere interface (as a covalent fusion to a specific inner kinetochore protein), the sensor was constitutively phosphorylated, regardless of whether tension was present or not. On the other hand, a sensor positioned near the outer plate of the kinetochore, again as a covalent fusion, was differentially phosphorylated in a manner that correlated with the degree of tension at the kinetochore [100]. These observations strongly suggest that in the absence of tension, Aurora B is within the reach of its kinetochore substrates, while rising levels of intra-kinetochore stretch progressively impair its ability to reach such substrates [22,40,90,91].

This idea is consistent with previous models suggesting that stabilization of kinetochore–microtubule interaction might be a consequence of the increasing separation of substrates from the destabilizing activity of Aurora B as attachment proceeds [50]. In its original formulation, however, this hypothesis predicted that tension separated a centromeric pool of Aurora B from its substrates at the kinetochore. The new data, on the other hand, suggest that the distance between Aurora B and its substrates increases by approximately 35 nm or less, i.e. by a size that might be similar or even larger than the size of the CPC itself. It is very unlikely that tension-sensitive differences in the shape of a diffusible gradient of Aurora B or of an Aurora B substrate can be established within the nanometre-scale structural deformations associated with intra-kinetochore stretch.

These considerations force us to predict that there is a kinetochore-associated pool of Aurora B. The function of this pool, which is highly relevant to the phenomena discussed here, probably is exercised on substrates that are located in close proximity to Aurora B in the absence of tension (possibly explaining why even little residual kinase activity is sufficient to maintain the checkpoint) and become separated when tension builds up. In other words, microtubule binding or force applied onto the kinetochore might result in the relative movement of molecular parts whose length scale has the same range as the extent of stretch. For instance, we have recently proposed a ‘ruler’ or ‘dog-leash’ model to explain how the Aurora B sensor might work [22]. The dog-leash model proposes that the distance to which Aurora B (the dog) can travel away from the base of the kinetochore (the dog's owner, represented by the Survivin and Borealin subunits) is limited by the maximal stretch of the leash, the INCENP subunit of the CPC. The dog-leash hypothesis remains completely speculative. Its rigorous testing will require a deeper understanding of the molecular mechanisms through which Aurora B phosphorylation of substrates influences the error correction and checkpoint responses.

## 6. Identification of the kinetochore receptors of checkpoint components

At this time, we have only a vague idea of the exact mechanism through which Aurora B controls the behaviour of its substrates. Aurora B phosphorylation of a motif in the N-terminal region of the kinetochore subunit KNL1/Spc105 prevents the recruitment of protein phosphatase 1 (PP1), probably through steric hindrance or neutralization of the positive charge of the Aurora B consensus site [101]. A similar principle might apply to the phosphorylation of the N-terminal tail of Ndc80, which, together with other segments of the Ndc80 protein [102,103], provides a crucial contribution to the microtubule-binding activity of the kinetochore [79,104]. In the above-mentioned cases, Aurora B phosphorylation negatively regulates interactions of its substrates with target proteins. On the other hand, Aurora B can also promote protein interactions at the kinetochore. For instance, it promotes the recruitment of several SAC proteins, including Mps1, to kinetochores [69,77]. Whether this phosphorylation-dependent recruitment reflects the existence of protein domains capable of binding the phosphorylated Aurora B consensus site is currently unknown.

In general, the identification of phosphorylation events that are relevant to the recruitment and activation of the components of the checkpoint and error-correction pathways are crucial tasks for future studies. Ideally, checkpoint-relevant substrates of Aurora B should be phosphorylated under conditions of low intra-kinetochore stretch, and become progressively dephosphorylated upon increasing stretch [105]. If the phospho-epitopes that promote kinetochore recruitment of the checkpoint proteins were ‘transplantable’ to other kinetochore proteins or to different regions of the same protein, testing the effects of re-positioning the phospho-epitopes relative to the predicted position of Aurora B will become possible. ‘Transplantability’ implies that kinetochore regions that are necessary and sufficient for the interaction with a given checkpoint protein are identified and characterized. What are the challenges associated with this task?

With the exclusion of the Aurora B-containing CPC, which is recruited to H3-containing nucleosomes, as discussed above, all additional receptor sites for the checkpoint components are probably contained within the 10-subunit KMN network [1]. For instance, the RNAi-based depletion of subunits of the Ndc80 complex prevents kinetochore association of the checkpoint proteins Mad1, Mad2 and Mps1 and of the subunits of the RZZ complex [27,106]. Conversely, Knl1 has been implicated in kinetochore recruitment of Bub1 and BubR1 [107]. Although these observations clearly point to the KMN network as a necessary scaffold for checkpoint signalling, the minimal structural elements required for high-affinity binding of the checkpoint components have so far escaped identification. To date, the only checkpoint-relevant interaction whose minimal requirements have been identified and recapitulated *in vitro* is the recruitment of O-Mad2 by the Mad1–C–Mad2 complex via conformational dimerization [108,109].

At least in part, difficulties in the identification of the binding sites of the checkpoint proteins are a consequence of the transient nature of their interaction with mitotic kinetochores. For instance, the Mps1 kinase turns over rapidly at kinetochores, suggesting that its interaction with kinetochores is of relatively low-affinity and possibly difficult to capture with classical methods for studying protein interactions within cells [110]. Another SAC protein, Mad1, displays relatively long residency times at mitotic kinetochores [110,111]. Yet, this protein is refractory to co-immunoprecipitation with other kinetochore proteins [27], possibly a reflection of cooperative binding through multiple low-affinity interactions, made possible, at least in part, through Mad1 oligomerization. These low-affinity interactions may not resist kinetochore solubilization and dilution prior to immunoprecipitation, leading to complex dissociation.

Additional difficulties in the identification of minimal binding domains might stem from the complexity of the multi-subunit interacting samples. We suspect that at least in part, these problems will be alleviated through work on biochemical reconstitution. With the availability of sufficient amounts of purified recombinant or native kinetochore samples [79,89,92,93,112], work on *in vitro* reconstitution might be expected to contribute to the identification of the precise mechanisms of kinetochore recruitment of the checkpoint proteins.

## 7. Checkpoint silencing

There has been considerable recent progress on the identification of the mechanism of checkpoint silencing. An interesting area of research focuses on the role of Cdk1 : Cyclin B activity in the checkpoint response and its suppression. As cells enter metaphase from prometaphase, they satisfy the checkpoint despite the presence of high Cdk1 activity. Checkpoint inactivation under these conditions is reversible: checkpoint silencing can be reverted if a spindle-damaging agent is added even after the beginning of Cyclin B degradation [113]. If Cyclin B destruction is prevented, error correction and checkpoint signalling are re-instated upon entry into anaphase [114,115]. To render checkpoint inactivation irreversible and to prevent error correction, inhibition of Cdk1 : Cyclin B might be required [116,117]. Thus, although Cdk1 : Cyclin B activity might be necessary for checkpoint signalling [118], reversible checkpoint inactivation is possible in the presence of high Cdk1 activity, suggesting that downregulation of Cdk1 activity is not necessary for checkpoint silencing but only to prevent checkpoint reactivation.

The PP1 phosphatase has emerged as a crucial antagonist of Aurora kinase activity at the kinetochore [72,75,101,119]. In the presence of spindle damage, PP1 activity is required to enforce mitotic exit when Aurora B activity is artificially repressed [72,75]. The emerging theme is that Aurora B antagonizes the localization of PP1 catalytic subunits through direct phosphorylation of motifs, conforming to the consensus sequence RVXF (where X may be one of several amino acids, but not a negatively charged or a phosphorylated one), which mediate PP1 localization [120]. The function of two such motifs, in the Knl1 and CENP-E kinetochore proteins, has been recently discussed [101,119]. By acting as PP1 docking sites and as targets of Aurora B, these motifs might contribute to promoting bi-stability in the checkpoint response. If the emerging picture is that Aurora B and PP1 form an antagonistic pair, how the activity from the other checkpoint kinases is reverted is currently unclear.

Another important area of research concerns the mechanism of microtubule-dependent removal of checkpoint proteins from kinetochores [121]. The coiled-coil protein Spindly has recently emerged as a crucial link between the RZZ complex and Dynein, the minus-end-directed motor required for removal of checkpoint proteins to kinetochores [122–125]. The RZZ is crucially required for the recruitment of the Mad1 : Mad2 complex to unattached kinetochores [126]. Like Mad1 : Mad2, the RZZ is transported away from kinetochores in a dynein-dependent manner in a process known as kinetochore ‘stripping’. Retention of Mad1 : Mad2 at fully attached kinetochores through its covalent fusion to a subunit of the Mis12 complex is sufficient to provoke a metaphase arrest [36]. A virtually identical phenotype is observed upon expressing a mutant of Spindly that prevents Mad1 : Mad2 re-localization owing to its inability to interact with Dynein [123]. These studies indicate that retention of the Mad1 : Mad2 complex at attached kinetochores is a sufficient condition for continued checkpoint signalling, so that the removal of this complex from kinetochores is necessary for checkpoint silencing.

An implication of the observations discussed in the previous paragraph is that once it becomes removed from kinetochores, the Mad1 : Mad2 complex might become inactivated and unable to signal to the checkpoint. Precisely how this happens is currently unclear. It is possible that Mad1 : Mad2 forms a ternary complex with a checkpoint antagonist known as p31^comet^, a protein that is structurally related to Mad2 and that binds to the closed conformer of Mad2 (C-Mad2) [127–130]. As a C-Mad2 binder, p31^comet^ has the potential to play a dual function in checkpoint silencing as a binder of C-Mad2 in the Mad1 : Mad2 complex or in the MCC [131,132]. Future studies will have to unravel the relative importance of these checkpoint-inactivating functions.
